# Continued norovirus false positivity after FDA clearance of the BioFire FilmArray gastrointestinal panel

**DOI:** 10.1128/spectrum.02990-25

**Published:** 2026-03-30

**Authors:** Michael Daley, Melinda D. Poulter, Emily A. Snavely

**Affiliations:** 1 UVA Clinical Laboratories, Microbiology and Molecular Infectious Diseases, UVAHealth, Charlottesville, Virginia, USA; 2 Division of Laboratory Medicine, Department of Pathology, University of Virginia School of Medicine 12349, Charlottesville, Virginia, USA; ARUP Laboratories, Salt Lake City, Utah, USA

**Keywords:** false-positivity, gastrointestinal Illness, norovirus

## LETTER

Rapid detection of enteric pathogens is a core responsibility of the clinical microbiology laboratory. Syndromic panels like the BioFire FilmArray Gastrointestinal Panel (BF-GIP) (bioMérieux, USA) provide PCR-based detection of multiple bacterial, parasitic, and viral pathogens in about 1 h (1, 2). While most enteric infections are self-limiting, false-positive results can lead to unnecessary treatment and delays in addressing alternative diagnoses.

In January 2024, bioMérieux voluntarily recalled the BF-GIP due to increased reports of false-positive Norovirus GI/GII results and recommended confirmatory testing when results were inconsistent with clinical presentation. A revised 510(k) application was cleared by the FDA in November 2024, after which many laboratories resumed BF-GIP testing without confirmatory workflows. We share our single-center evaluation of norovirus performance following recall resolution.

Beginning March 2024, norovirus-positive BF-GIP results deemed clinically questionable by the ordering clinician were sent to Mayo Clinic Laboratories for confirmatory testing. In May 2024, confirmatory testing was expanded to include all positive results, and in September 2024, an in-house norovirus reflex protocol was implemented using the FDA-cleared BD MAX Enteric Viral Panel. Verification samples demonstrated 95.8% concordance between BD Max and the Mayo Clinic testing. Stool (Cary-Blair) and fecal eSwab specimens were tested within stability parameters, without significant delays or freeze–thaw cycles. Norovirus results were classified as true positives if confirmed by either method.

Between May 2024 and December 2025, norovirus was detected in 804 tests (14.2%), of which 532 (66.2%) were false positives, yielding a positive predictive value (PPV) of 33.8% and a negative percent agreement (NPA) of 90.1% (95% CI, 89.3–90.9%). False positives represented 9.4% of all BF-GIP tests, ranging from 3.1% in July 2024 to a peak of 19.9% in September 2024, and persisted throughout the study period without sustained decline after FDA re-clearance (3). Co-detection of at least one additional enteric pathogen occurred in 119 (14.8%) norovirus-positive BF-GIP tests, most commonly EPEC, EAEC, ETEC, *Campylobacter*, or other viral causes of gastroenteritis. These results represent 781 unique patients; 19 underwent repeat norovirus testing, with concordance across specimens collected a median of 39 days apart.

**Fig 1:**
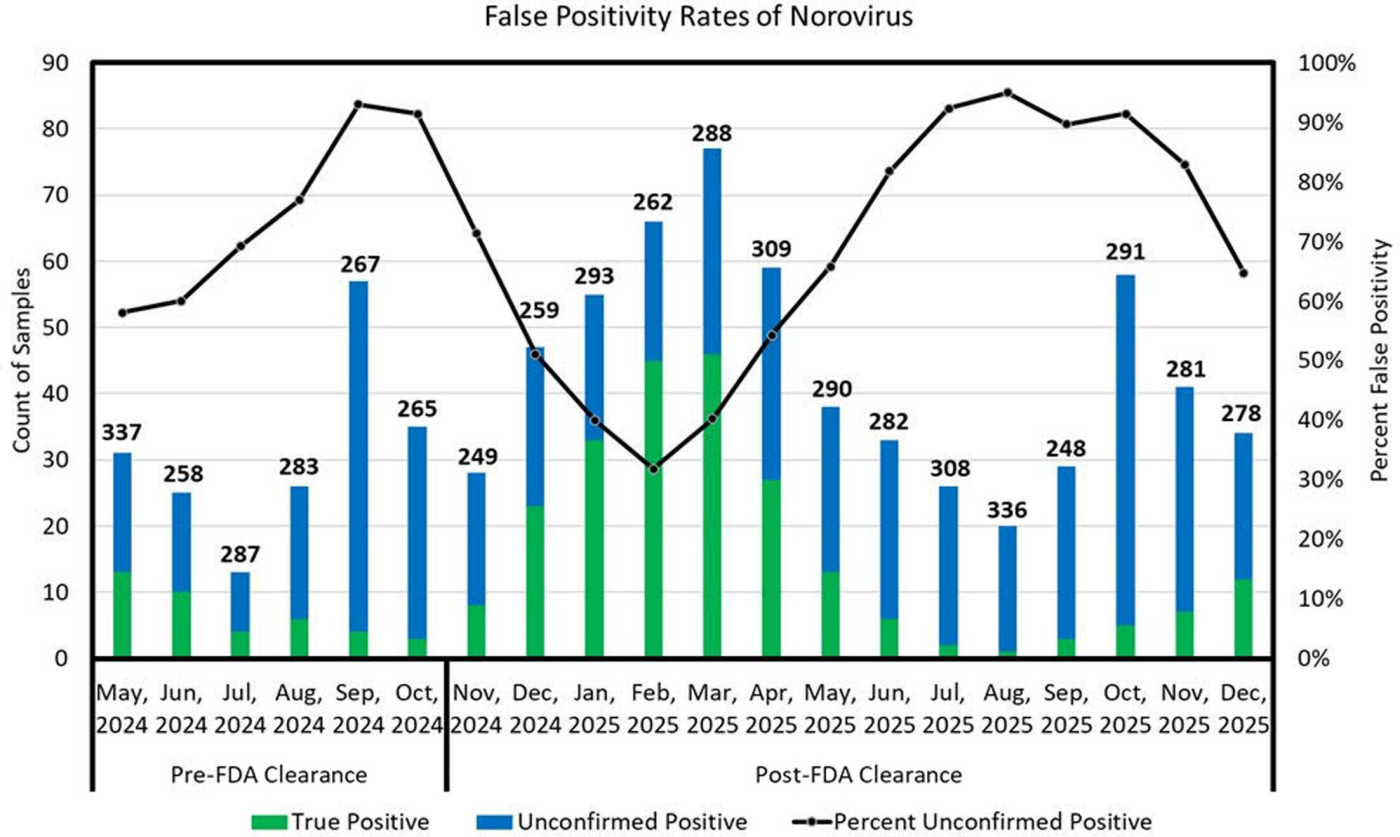
Distribution of false positive and true positive results for norovirus over our testing window. Divided based on recall status and FDA clearance. Numbers indicate total number of BF-GIP samples tested per month.

These findings diverge from the updated IFU, which reports an NPA of 96.5% (95% CI, 95.1–97.7%) and attributed false positivity to expanded cross-reactivity. In our cohort, false positivity remained elevated in the post-clearance period, peaking at 95% in August 2025 (Fig. 1), with a cumulative post-clearance rate of 62.19% (380/611).

This study is limited by its single-center design, limiting generalizability to other geographic locations. Confirmatory testing relied on assays with different limits of detection than the BF-GIP, which may contribute to discordant results at low viral loads. Although melt curve analysis was not performed (4), BF-GIP crossing point (Cp) analysis demonstrated increased unconfirmed positivity at higher CP values, consistent with detection near the assay limit. However, this pattern alone does not explain the magnitude or persistence of false positivity observed. Despite these limitations, the frequency of false-positive norovirus detection after FDA clearance remains concerning and is not sufficiently addressed in the IFU addendum.

Given these findings, laboratories should consider continued confirmatory testing of BF-GIP norovirus results until further refinements reduce false positivity. Returning to pre-recall practices may pose risks to patient care.
